# Supplementary material for: Synergistic Hypolipidemic Effects and Mechanisms of Phytochemicals: A Review
Source: Foods. 2022 Sep 9;11(18):2774. doi: 10.3390/foods11182774 (PMC9497508; doi:10.3390/foods11182774)
Supplement: Supplementary file 1 [file foods-11-02774-s001.zip › foods-1881328-supplementary.pdf]

Supplementary material:

Figure S1.

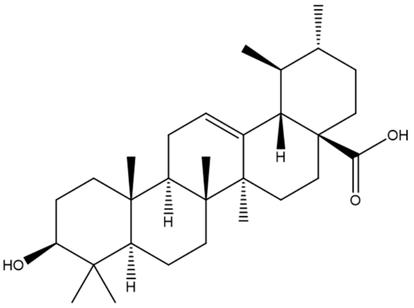

ursolic acid

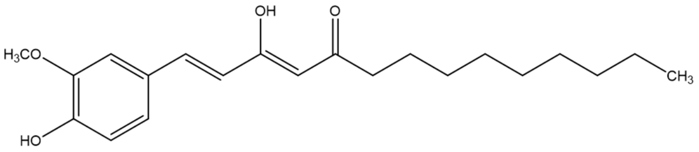

10-dehydrogingerdione

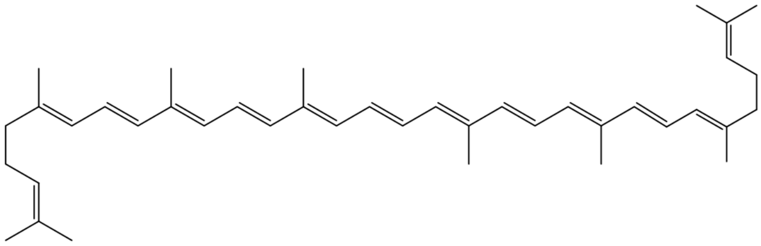

lycopene

Figure S1. Basic structures of other phytochemicals that have been found to have possible synergistic hypolipidemic effects.
